# Longitudinal recordings of the vocalizations of immature Gombe chimpanzees for developmental studies

**DOI:** 10.1038/sdata.2014.25

**Published:** 2014-08-19

**Authors:** Frans X. Plooij, Hetty van de Rijt-Plooij, Martha Fischer, Anne Pusey

**Affiliations:** 1 International Research-institute on Infant Studies, 6814 CE, Arnhem The Netherlands; 2 Macaulay Library, Cornell Lab of Ornithology, Ithaca, New York 14850, USA; 3 Department of Evolutionary Anthropology, Duke University, 27708 Durham, North Carolina, USA

## Abstract

Many researchers are interested in chimpanzee vocal communication, both as an important aspect of chimpanzee social behavior and as a source of insights into the evolution of human language. Nonetheless, very little is known about how chimpanzee vocal communication develops from infancy to adulthood. The largest dataset of audiorecordings from free-living immature chimpanzees was collected by the late Hetty van de Rijt-Plooij and Frans X. Plooij at Gombe National Park, Tanzania (1971–1973). These recordings have not yet been analysed. Therefore, the most extensive effort to study the development of chimpanzee vocalizations remains unfinished. The audiospecimens total over 10 h on 28 tapes, including 20 tapes focusing on 17 specific immature individuals with a total of 1,136 recordings. In order to make this dataset available to more researchers, the analogue sound recordings were digitized and stored in the Macaulay Library and the Dryad Repository. In addition, the original notes on the contexts of the calls were translated and transcribed from Dutch into English.

## Background & Summary

Chimpanzees resemble humans in many respects^1^, but differ greatly from us in terms of vocal communication. One of the most striking ways in which human language differs from the vocal communication of our primate relatives is the extent to which vocal signals are learned^2^, rather than innate. While songbirds^3–5,3–5,3–5^, some whales^6–11,6–11,6–11,6–11,6–11,6–11^, and even elephant seals^12^ show evidence for vocal learning most mammals, including nonhuman primates, have shown little evidence for learning^13^ (for exceptions see refs 14,15) raising questions about how and why this trait arose in the human lineage^5^. While captive chimpanzees can learn to use some elements of human language, they cannot effectively imitate speech, and their natural vocal communication shows little evidence of language-like features^16^.

Recent studies, however, have found intriguing evidence of aspects of chimpanzee vocal communication that resemble features of human language, such as referential signaling^17,18^ and regional dialects^19–21,19–21,19–21^. If dialects cannot be explained simply by regional differences in ecology^22^ their existence is particularly intriguing, as it suggests that chimpanzees are capable of at least some learned modification of the acoustic structure of their calls. Unfortunately, no study has yet examined how the acoustic structure of these calls changes over the life of an individual, a crucial question for determining whether differences result from vocal learning. Documenting such changes is particularly difficult in chimpanzees, which live long and mature slowly. To date, very little research has been done on the development of chimpanzee vocal communication.

The most extensive effort to study the development of chimpanzee vocalizations remains unfinished. During their dissertation research on the development of behavior, the late Hetty H. C. van de Rijt-Plooij and her husband Frans Plooij recorded vocalizations of chimpanzees in Gombe National Park, Tanzania, from 1971–1973 in combination with notes on their direct observations of chimpanzee behavior and other contextual information surrounding the vocalizations. The couple focused on recording younger individuals but also recorded individuals of all ages. However, having collected a wealth of other data in the field as well, these authors published on the behavioral development of chimpanzee infants and the development of the mother-infant relationship^23–36,23–36,23–36,23–36,23–36,23–36,23–36,23–36,23–36,23–36,23–36,23–36,23–36,23–36^ and did not analyze the vocalizations.

The centrepiece of the data package described in this paper is a collection of sound recordings of vocalizations of infant, juvenile and adolescent chimpanzees now available from the Macaulay Library (Data Citation 1) with extensive metadata (see section ‘Data Records’) for each recording. Supplementary data files are available from Dryad (Data Citation 2). All individuals were recorded longitudinally for nearly 2 years. Table 1 presents the names, birth dates, age class, sex, span of longitudinal recordings in months, and the number of recordings for each individual. The total number of recordings is 1,136. The remaining recordings concern an orphaned chimpanzee infant named Kobi, who was born in what is now the Democratic Republic of Congo and who was temporarily raised by humans in the Gombe National Park camp; he was not part of the Gombe chimpanzee community. For more information see ref. 37.

In addition to the recordings collected by van de Rijt and Plooij (1971–1973), recordings of the same population also exist made by Peter Marler in 1967^38–40,38–40,38–40^, Charlotte Uhlenbroek in 1991–1993^41^, and Lisa O’Bryan in 2009–2010 (Ph.D., candidate University of Minnesota). However, these recordings mainly concern adult individuals. Some of these adults were also recorded as infants/juveniles in the period 1971–1973, though, and comparing their adult recordings with their infant/juvenile recordings might be an especially effective way of studying vocal development.

Our recordings and behavioral observation notes will be useful to researchers interested in comparative studies and a variety of questions relating to the development, contexts, and bioacoustics of chimpanzee vocal communication. For example, McCune and coworkers^42–44,42–44,42–44^ suggest that grunt communication is a developmental phase in human infants with evolutionary significance. Our data set could be used in a study comparing the grunts of human and chimpanzee infants.

## Methods

**Figure 1:**
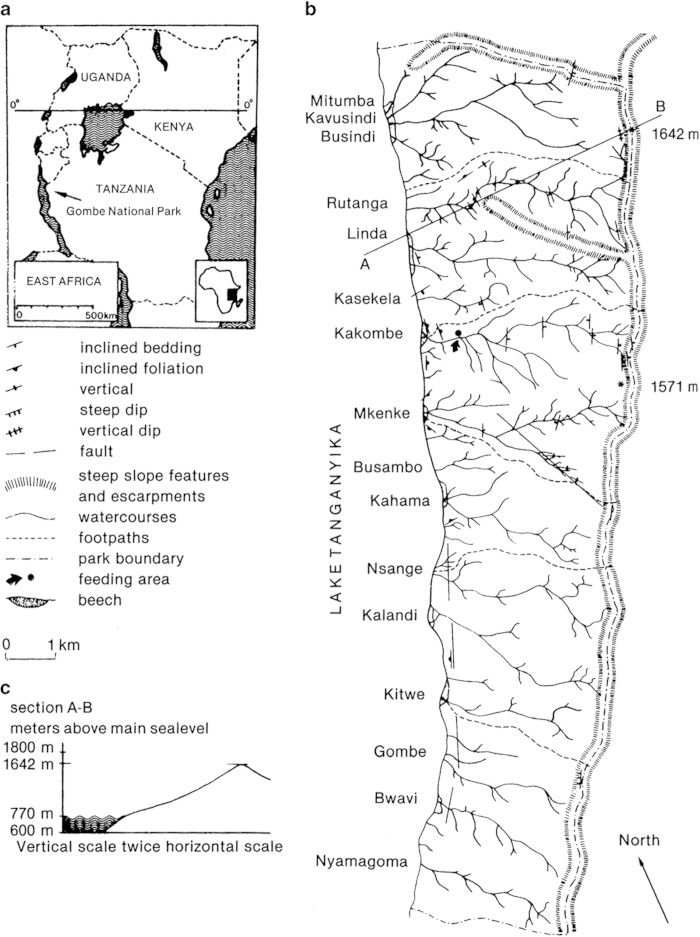
Situation and map of the Gombe National Park (from Plooij^27^, with permission of the publisher). (**a**) Map of East Africa indicating the location of the Gombe National Park in Tanzania. (**b**) The general structure of the Gombe National Park. (**c**) West-east profile of the park (along line A-B in Figure 1b).

**Figure 2:**
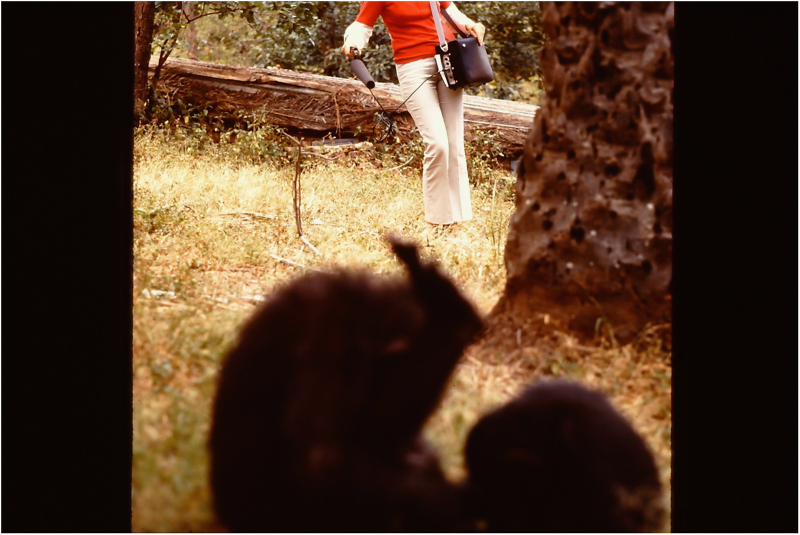
The recording situation of the chimpanzee vocalizations in the Kakombe valley in the feeding area (see Figure 1b). This was the open place where Jane Goodall fed the chimpanzees. At a distance between 5–15 meters the recordist (red sweater) was carrying a Nagra sound recorder (full track mono, 19 cm/s) and pointed a Sennheiser MKH 815T directional microphone (covered with a windhose) at the chimpanzees (shaded figures in the foreground). Photo copyright Frans X. Plooij.

A map of the Gombe National Park, Tanzania, East Africa is shown in Figure 1. Most recordings were made in the feeding area located in the Kakombe valley (see Figure 1b). This was the open place where Jane Goodall fed the chimpanzees^1^. The recording situation there was as follows. At a distance between 5–15 m the recordist pointed a Sennheiser MKH 815T directional microphone (covered with a windscreen) at the chimpanzees (shaded figures in the foreground of Figure 2) while carrying a running Nagra sound recorder (full track mono, 19.05 cm/s or 7.5 inch/s). Apart from the chimpanzee vocalizations, the verbal commentary of the recordist was recorded, before or after the vocalizations. The verbal commentary covered the names of the chimpanzees and the names of the vocalizations they produced, together with a description of the behaviour surrounding the vocalizations. Definitions of the chimpanzee behavior categories, illustrated with drawings by David Bygott, were published by Plooij^27^ in Appendix A of his book.

After the sound recordings were made, analogue audio specimens were selected from the tape and coupled with metadata that consisted of the transcriptions of the verbal commentary in Dutch and a number of other information items that are described under Data Records. The analogue audio specimens were created by listening to the original recordings and cutting out the stretches of tape containing chimpanzee vocalizations. The stretches of tape were glued together and stored on 28 reels totalling 10 h of chimpanzee vocalizations, where 20 reels concerned 17 specific young individuals (one (or more) separate reel for each individual) and 8 reels concerned adult individuals.

In 2010 the analogue audio specimens were digitized and provided together with the audiotapes to the Macaulay Library. The transcriptions of the verbal commentary were translated from Dutch to English. These transcriptions and associated metadata (see Data Records) were entered into spreadsheets (one per individual) and then into the Macaulay Library database (see Data Citation 1).

## Data Records

**Figure 3:**
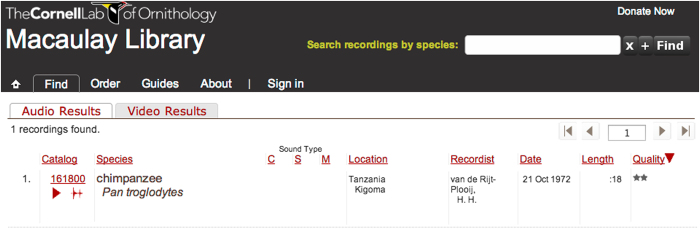
A screenshot of a Macaulay Library website search result. Clicking on the Macaulay Library Catalog number (i.e., 161,800) will take the reader to an automatic playing of the audio along with the recording’s full set of metadata. Clicking on the red triangle plays the audio; clicking on the waveform icon brings up the audiofile in RavenViewer.

The 1,248 audiospecimens at the Macaulay Library can be accessed directly via Data Citation 1 or by searching for ‘chimpanzee’ and ‘*Pan troglodytes*’ recordings with ‘Van de Rijt-Plooij, H.’ as the recordist (see Figure 3). Each record, which can be played back online, includes the following metadata: the catalog number, the species name, the recording date, the recording geography with map, the latitude/longitude, the media and equipment used, the name of the recordist, the recording length (duration), recording quality (rated according to a five star system) and notes. ‘Recording Quality’ indicates the signal-to-noise ratio with 5 stars meaning clear vocalization and very low noise in the recording. For a further specification of the measurement behind the 5 star system, see the Technical Validation section. Notes include the names of the vocalizing individual(s) together with the vocalization(s) of each individual and the behaviour and situation surrounding the vocalizations. Many recordings contain multiple calls by multiple animals. This means the sample size is overall quite large.

Table 2 summarizes the number of each type of vocalization given by each focal individual or the other individuals also vocalizing during the recordings of that focal individual. This table gives an indication of the frequency of the various call types. In the counting process for this summary, no discrimination was made between the calls of the focal individual and the calls of the other individuals, who were also vocalizing during the recordings of the focal individual but the identities of the callers can be distinguished in Metadata table (see below). Therefore, the top part of the table corresponds with the list of infant vocalizations and the bottom part contains 7 call types that are typically given by older individuals. It is striking that the total frequencies of the call types ‘Grunt’, ‘Ho’, ‘Hoo’, ‘Hoocall’, ‘oo’, and ‘Tonalgrunt’ are quite high. This is promising for a study comparing the grunts of human and chimpanzee infants.

Metadata for all the immature individuals, cross-referenced by Macaulay catalog number, have been submitted to Dryad (Data Citation 2) in order to allow users to search for specific recordings beyond the capabilities currently provided by the Macaulay Library web interface. The first file of these metadata is a spreadsheet (All Infants_3July2014final.xlsx) and includes the name(s) of the vocalizing individual(s), the vocalization, the behaviour, and other details. The first column of the spreadsheet contains the Macaulay catalog number and that is the link to the Macaulay database. The spreadsheet is basically the same as the Macaulay database except that the columns are organized in a slightly different way. From left to right the following columns can be found: ‘Macaulay catalog number’, ‘Recording Device’, ‘Focal individual’, ‘Focal Birthdate’, ‘Recordist record number’, the ‘Level of Recording’ as selected on the Nagra sound recorder, the ‘Quality outstanding’ column where an x indicates a recording that is outstanding for various reasons (such as a very clear, good-quality recording, a recording where the vocalization is without other, simultaneous vocalizations, a recording that is a good example of the development of infant vocalizations, a recording showing nice mother-infant interaction, a recording that illustrates how the infant ‘follows’ the vocalizations of the mother), the ‘Month’, ‘Day’ and ‘Year’ of the recording, the ‘Other individuals Vocalizing’ in the recording of the Focal Individual, the ‘Date’ of the recording, the ‘Focal age’ (the age in years of the focal individual at the date of recording), the ‘Individual(s) with sound/call type’, the ‘Observation of context’ and behaviors surrounding the vocalizations, the ‘Macaulay Library Public Notes’ field, the ‘Microphone’, the ‘Recorder’, and the ‘Tape Speed’. As is described in the Usage Notes section, the grammar of the column containing individual(s) with sound/call type is such that the sequence of vocalizing is preserved. This gives information on who initiated calling, if several individuals called. This is important because it shows that vocalizations of others often triggered infants to vocalize. In the column ‘Observation of the context and behaviors surrounding the vocalizations’ the presence of nearby individuals was also noted, even if they did not vocalize.

Furthermore, the Dryad data package includes the unparsed digital copies of the chimpanzee tapes (the source analog reel-to-reel media that the Macaulay Library converted to 96 kHz/24-bit files) and two additional data files. One file is the Gombe_biography (Gombe_biography-for_1971-3.xls) for the chimpanzee individuals present during the span of time that the recordings were made. The Gombe biography contains 13 columns, but the ones most useful for analyzing the vocalizations are the name of the individual (column B), the birth date (column C), the name of the mother (column H) and the sex of the individual (column I). These columns are selfexplanatory. The other columns are explained in Strier *et al.* ^45^ for those who are interested in them. The second file is a selfexplanatory list of names of infant vocalizations (List of names of infant vocalizations.doc) as used in the spreadsheet (All Infants_3July2014final.xlsx) and the Macaulay database.

## Technical Validation

**Figure 4:**
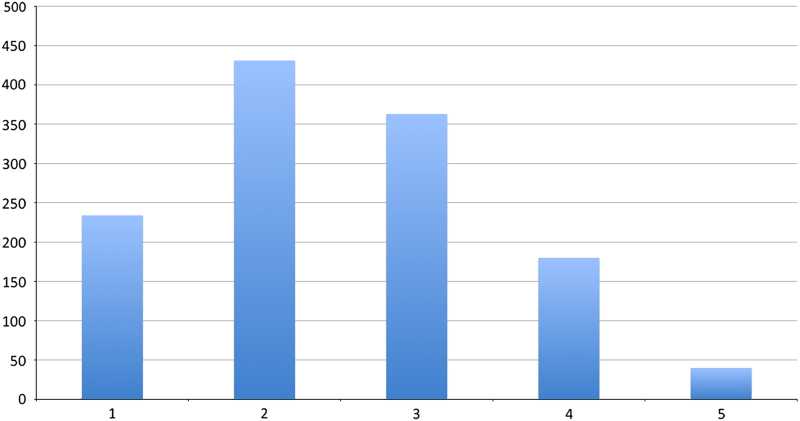
The frequency distribution of the absolute number of recordings (*y*-axis) over the ratio of signal power to noise power (SNR) expressed in number of stars (x-axis). The average SNR over 1,248 recordings is 2.49.

The ‘Quality’ of the soundrecordings in the Macaulay Library is an informal and rough Indication of the ratio of signal power to noise power (SNR). Five stars means that the recording has an SNR of 50:1 (3.2% of the 1,248 recordings were given this rating; four stars means an SNR of roughly 40:1 (14.4%); three stars conveys an SNR of roughly 30:1 (29.0%); two stars points to an SNR of roughly 20:1 (34.6%); and one star indicates SNR of less than 10:1 (18.8%)). The frequency distribution of the absolute number of recordings (*y*-axis) over the ratio of signal power to noise power (SNR) expressed in number of stars (*x*-axis) is given in Figure 4.

Nearly all the sound recordings were collected by Hetty van de Rijt-Plooij.

It was impossible to test inter-observer reliability in the field, because it was difficult for two observers to be both in the best observing spot. However, intra-observer reliability can be tested over repeated observations and transcriptions of the same recordings. For example, for a short period during our study there was a portable video-recorder available in Gombe, together with a monitor. One of the infants was videotaped for 13 min during an episode which was as ‘difficult’ as possible: A number of other individuals were present and the focal infant was playing with another infant. We would expect this episode to represent a worst case for intra-observer reliability.

The intra-observer reliability test was done on two successive evenings speaking a verbal commentary onto an audiotape in terms of a list. The transcriptions of the audiotapes were done with an interval of one week between successive transcriptions. The analysis was done as follows:

First, an overall measure of reliability was calculated. The total number of behaviour category combinations was counted. (If two or more behaviour categories occur simultaneously, these form a combination.) This number depends not only on the behaviour-sequence of the infant but also on the behaviour changes in the other individuals. Therefore, it is quite a sensitive measure. According to this measure, intra-observer reliability is high: the first transcription had 215 combinations versus 217 for the second. This gives a reliability of 0.99.

Second, the infant-behavior-sequence of the first test was compared with the one of the second test. For every 15-second period the frequency of newly started behaviour categories was established. Thereafter, the behaviour categories of the two corresponding 15-second periods were compared and the number of behaviour categories that were the same were counted. Numbers of all the 15-second periods were added together and the total was called S. The total number of behaviour categories (S+the other categories that were dissimilar) was taken as the sum of those 15-second period frequencies which had the highest number of units (T). The intra-observer reliability (R) was calculated according to the formula: R=100×S/T. The result of 76% is deemed to be acceptable^46,47^ as the test had been made as difficult as possible and considering the minor/subtle mistakes made. The cause for the dissimilarities was roughly threefold.

First, in many cases the sequence was the same, except that in one test a behaviour category combinations was transcribed just before a 15-second time marker and just after the same marker in the second test. The reason for this was that the verbal commentary lagged behind a little when a lot happened at once and, furthermore, this lagging behind varied from test to test.

Second, there are some examples where the categories in test 1 were dissimilar but related to the categories recorded in test 2. For instance, ‘looks at’ versus ‘glimpse’. Here a judgement of the duration is crucial and it is understandable that mistakes are made in marginal cases.

Third, behaviour categories were recorded in one test and missed in the other. Mostly, these were short-lasting categories such as glimpse, yawning, self-scratching, shaking. Taking these kinds of mistakes into consideration we feel confident that the intra-observer reliability is high enough to use the data for further analysis.

## Usage Notes

The age of the focal individuals at the moment of a recording was calculated by subtracting the date of birth (taken from the Gombe biography file) from the date of recording.

‘Individual(s) with sound/call type’ (Column N of the metadata spreadsheet ‘All Infants_3July2014final.xlsx’) gives the names of all vocalizing individuals together with the vocalization(s) they produce. A note of ‘uncertain’ behind a name means the recordist is not quite sure the vocalization came from that individual; ‘UN’ means ‘unknown individual(s)’; ‘GEN’ means ‘General’ or ‘the whole group’; ALL means all individuals present; HUM means ‘human’; BAB means ‘baboon’. The names plus vocalization are separated by ‘,‘ (comma). This column makes ‘cross-references’ superfluous. The Grammar of column N is as follows:

- A comma followed by a single space separates vocalizations following each other immediately, or between the last vocalization of one individual and the name of the next individual in the sequence. All the vocalizations between two names belong to the individual of the first name.
- ‘…’ indicates that some time passes by between one vocalization and the next.
- Parenthical comments, such as ‘(huu)’, which is a Dutch dipthong, ‘(hoo)’, ‘(soft)’ or other remarks after the name of the vocalization describes how the vocalization sounds or gives a qualification or a general remark concerning the sound or the recording process. Whenever it says: ‘recording needle trembling’, the literal translation of the original note would be ‘recording knob shaking’. However, because we do not understand how such a knob can shake, it is translated instead as ‘needle trembling’.
- ‘General’ means: the whole group.

In Column O (‘Observation of Context’) of the metadata spreadsheet ‘All Infants_3July2014final.xlsx’ a more general behavioural context is given of the vocalizations involved in the recording. Whenever numbers are used, these refer to the distance categories as defined on page 24 of Plooij^27^.

## Additional information

**How to cite this article:** Plooij, F. X. *et al.* Longitudinal recordings of the vocalizations of immature Gombe chimpanzees for developmental studies. *Sci. Data* 1:140025 doi: 10.1038/sdata.2014.25 (2014).

## Supplementary Material



## Figures and Tables

**Table 1:** The names, birth dates, age class, sex, span of longitudinal recordings in months, and the number of recordings for each chimpanzee individual recorded in Gombe National Park in the period 1971–1973.

**Animal name**	**Birth date (approximately)**	**Age class**	**Sex**	**Age span longitudinal recordings (months)**	**Number of recordings**
WILKIE	21-oct-72	Infant	M	0–3	32
PROF	26-oct-71	Infant	M	0–15	183
BEE HINDE	15-jun-71	Infant	F	0–19	0
FREUD	22-may-71	Infant	M	0–20	248
GREMLIN	19-nov-70	Infant	F	7–27	55
PLATO	07-sep-70	Infant	M	10–29	124
SKOSHA	27-mar-70	Infant	F	14–30	96
MOEZA	20-jan-69	Infant	F	28–50	60
ATLAS	25-sep-67	Infant	M	43–65	10
MUSTARD	22-nov-65	Juvenile	M	66–88	37
POM	13-jul-65	Juvenile	F	70–92	25
GOBLIN	06-sep-64	Juvenile	M	80–102	55
FLINT	01-mar-64	Juvenile	M	86–108	114
SHERRY	02-jul-61	Adolescent	M	118–140	43
GILKA	02-jul-60	Adolescent	F	130–152	37
LITTLE B	02-jul-60	Adolescent	F	130–152	3
SNIFF	02-jul-59	Adolescent	M	142–164	14
FIFI	02-jul-58	Adult	F	NA	
WINKLE	02-jul-58	Adult	F	NA	
MIFF	02-jul-56	Adult	F	NA	
JOMEO	02-jul-56	Adult	M	NA	
SATAN	02-jul-55	Adult	M	NA	
GIGI	02-jul-54	Adult	F	NA	
FIGAN	02-jul-53	Adult	M	NA	
GODI	02-jul-53	Adult	M	NA	
NOVA	02-jul-53	Adult	F	NA	
EVERED	02-jul-52	Adult	M	NA	
PALLAS	02-jul-52	Adult	F	NA	
ATHENA	02-jul-52	Adult	F	NA	
CHARLIE	02-jul-51	Adult	M	NA	
NOPE	02-jul-50	Adult	F	NA	
WILLY WALLY	02-jul-49	Adult	M	NA	
MANDY	02-jul-49	Adult	F	NA	
MELISSA	02-jul-49	Adult	F	NA	
PASSION	02-jul-49	Adult	F	NA	
DE	02-jul-48	Adult	M	NA	
FABEN	02-jul-47	Adult	M	NA	
HUMPHREY	02-jul-46	Adult	M	NA	
MADAM B	02-jul-45	Adult	F	NA	
HUGH	02-jul-44	Adult	M	NA	
MIKE	02-jul-38	Adult	M	NA	
GOLIATH	02-jul-37	Adult	M	NA	
HUGO	02-jul-36	Adult	M	NA	
FLO	02-jul-19	Adult	F	NA	
Span of longitudinal recordings in months, and the number of recordings are only given for the immature individuals. The total number of recordings is 1,136. The remaining recordings concern an orphaned chimpanzee infant named Kobi, who was born in what is now the Democratic Republic of Congo and who was temporarily raised by humans in the Gombe National Park camp; he was not part of the Gombe chimpanzee community. For more information see ref. 37.					

**Table 2:** Counts of call types by focal individuals.

	**Focal ID**																	
**Call type**	**Atlas**	**Flint**	**Freud**	**Gilka**	**Goblin**	**Gremlin**	**Kobi**	**Little B**	**Moeza**	**Mustard**	**Plato**	**Pom**	**Prof**	**Sherry**	**Skosha**	**Sniff**	**Wilkie**	**Grand total**
Bark	0	8	19	8	7	3	7	0	4	2	7	8	14	11	10	1	3	**112**
Beep	0	0	1	0	1	0	0	0	0	0	1	0	1	0	0	0	0	**4**
Burp	0	0	1	0	0	0	2	0	0	0	0	0	0	0	0	0	0	**3**
Choew	0	0	0	0	0	0	0	0	0	0	0	0	0	0	0	0	0	**0**
Crying	0	3	1	0	8	0	1	0	0	1	0	0	0	2	0	0	0	**16**
Developmentalpanthoot	0	0	0	0	0	0	0	0	0	0	0	0	0	0	0	0	0	**0**
Drumming	0	2	0	0	1	0	0	0	0	0	1	0	0	1	0	0	0	**5**
eagleraa	0	0	0	0	0	0	0	0	0	0	0	0	0	0	0	0	0	**0**
Effortgrunt	0	3	22	0	0	0	8	0	2	0	1	0	22	0	2	0	2	**62**
excitement	0	1	6	0	2	0	0	0	0	1	0	0	2	0	1	1	0	**14**
Flappinglips	0	0	0	0	0	0	0	0	0	0	0	0	0	0	0	0	1	**1**
groan	0	0	0	0	0	0	0	0	0	0	0	0	0	0	0	0	0	**0**
Grunt	2	42	83	25	14	16	32	1	11	5	44	8	73	21	24	10	10	**421**
Hiccup	0	0	0	0	0	0	1	0	0	0	1	0	1	0	0	0	0	**3**
Ho	5	23	32	3	13	12	16	0	17	11	23	5	41	18	20	5	9	**253**
Hoo	1	12	26	3	12	10	14	0	6	5	13	2	37	15	18	5	6	**185**
Hoocall	0	0	0	0	0	1	0	0	0	0	1	0	0	0	1	0	0	**3**
Hoot	1	6	20	3	10	6	2	0	1	4	6	2	10	14	14	5	5	**109**
Incipientpanthoot	0	0	2	0	0	0	0	0	0	0	0	0	0	0	0	0	0	**2**
Infantbark	0	0	2	0	0	1	2	0	0	0	1	0	8	0	5	0	0	**19**
Infanthoot	0	0	0	0	0	0	0	0	0	0	0	0	0	0	0	0	0	**0**
Infantsqueak	0	0	1	0	0	1	2	0	0	1	0	0	34	0	0	0	6	**45**
laugh	1	28	31	3	20	5	5	1	14	10	27	4	4	1	35	1	1	**191**
Laughing	1	26	7	3	20	3	5	1	11	10	26	4	1	1	33	1	1	**154**
Laughter	0	0	13	0	0	0	0	0	0	0	1	0	2	0	0	0	0	**16**
moan	0	0	2	0	0	0	0	0	0	0	0	0	0	0	0	0	0	**2**
nahts	0	0	0	0	0	0	0	0	0	0	0	0	0	0	0	0	0	**0**
oo	1	20	46	5	16	12	16	0	7	8	15	3	41	18	28	5	8	**249**
Pant	1	10	51	5	15	9	6	0	4	6	22	3	15	22	14	9	9	**201**
Pantgrunt	0	3	13	3	4	0	0	0	3	1	13	0	6	9	0	0	1	**56**
panthoot	1	2	17	2	8	6	1	0	1	4	5	1	6	14	11	3	5	**87**
Peep	0	0	0	0	0	0	1	0	0	0	0	0	1	0	0	0	0	**2**
Scream	0	24	8	4	12	1	11	1	8	6	4	10	2	6	10	0	4	**111**
Sigh	0	0	0	0	1	0	0	0	0	0	0	0	1	0	0	0	0	**2**
Silence	0	1	2	0	1	1	0	0	1	1	1	0	0	0	5	0	1	**14**
Sneeze	0	0	1	0	0	0	0	0	0	1	1	0	3	0	0	0	4	**10**
Softstaccato	0	0	20	0	0	4	8	0	0	0	1	0	4	0	0	0	5	**42**
Sound	0	4	38	1	3	3	2	0	8	1	5	0	8	4	15	0	2	**94**
Staccato	0	0	67	0	4	10	13	0	1	0	3	1	44	1	3	0	8	**155**
Stamping	0	4	7	0	5	1	0	0	0	0	3	0	0	0	6	0	0	**26**
Tonalgrunt	2	1	16	2	0	1	3	0	1	0	5	0	20	5	4	0	3	**63**
Uh	0	3	100	0	2	14	33	0	6	2	42	5	36	0	24	0	11	**278**
Uh_emphasis	0	0	7	0	0	2	0	0	0	0	0	0	1	0	0	0	0	**10**
Unknown	0	2	7	1	0	0	0	0	0	1	0	1	4	0	1	0	0	**17**
Waahbark	0	2	0	0	4	0	2	0	1	1	0	3	0	0	0	0	0	**13**
Whimper	4	15	12	0	16	8	32	0	23	11	16	2	22	6	4	0	2	**173**
Whimperho	0	1	0	0	0	1	1	0	0	0	0	0	0	1	0	0	0	**4**
Yawn	0	0	1	0	0	0	0	0	0	0	1	0	0	0	0	0	0	**2**
Foodbark	0	0	3	0	0	0	0	0	0	0	0	0	0	0	0	0	0	**3**
Foodgrunt	0	0	0	0	0	0	0	0	0	0	0	1	0	0	0	0	0	**1**
Pantbark	0	2	4	2	0	0	0	0	1	0	0	0	1	2	2	0	1	**15**
Pantscream	0	0	0	0	0	0	0	0	0	0	0	0	0	0	1	0	0	**1**
Squeak	4	14	5	7	7	2	2	1	8	4	5	2	36	4	7	2	6	**116**
Squeal	0	0	1	1	0	0	0	0	0	0	0	0	0	0	1	1	0	**4**
Cough	0	3	3	0	2	2	0	0	0	1	1	0	4	1	3	0	0	**20**
Infant-waah	0	0	0	0	0	0	0	0	0	0	0	0	0	0	0	0	0	**0**
**Grand Total**	**24**	**265**	**698**	**81**	**208**	**135**	**228**	**5**	**139**	**98**	**296**	**65**	**505**	**177**	**302**	**49**	**114**	**3,389**
In the counting process, no discrimination was made between the calls of the focal individual and the calls of the other individuals, who were also vocalizing during the recordings of the focal individual. Therefore, the top part of the table corresponds with the list of infant vocalizations and the bottom part contains 7 call types that are typically given by older individuals. The one but last row in the table concerns the call type ‘infant-waah’. This was added in the counting process to double check that this call type was never observed.																		
